# The use of magical plants by curanderos in the Ecuador highlands

**DOI:** 10.1186/1746-4269-5-3

**Published:** 2009-01-22

**Authors:** Anthony P Cavender, Manuel Albán

**Affiliations:** 1 Department of Sociology and Anthropology, East Tennessee State University, Johnson City, Tennessee 37604, USA; 2 Decano, Ciencias de la Salud, Universidad Estatal de Bolivar, Guaranda, Ecuador

## Abstract

Although the use of plants for treating supernaturally caused illnesses (e.g., soul loss, evil wind, witchcraft) has been documented in the Ecuador highlands, so-called magical plants have received much less focused attention than plants used for treating naturalistic disorders. Drawing on interviews done in 2002 and 2003 with 116 curanderos residing in the Ecuador highlands, this paper examines the characteristics of plants identified as magical, how they are used, and how the study of magical plants provides insights into the mindscape of residents of the highlands.

## Background

Traditional medical practitioners in the Ecuador highlands, those generically known as curanderos but especially the *limpiadores *("cleaners"), make extensive use of magical plants in the treatment of supernatural folk illnesses such as *susto*, *mal viento, mal prójimo*, and *mal aire*. In most documentations on magical plant use over the past thirty or so years, however, the rationale underlying the use of magical plants is poorly described or not addressed at all [1-5]. This paper reports on information gathered from 116 curanderos in the Ecuador highlands on the use of magical plants for healing. Topics discussed include the characteristics of plants identified as magical, why and how they are used for the treatment of selected folk illnesses, and finally how the study of magical plants provides insights into the worldview of highland residents.

## Methods

Residents of the Ecuador highlands have a wide variety of plants available for healing. Physiographically, the highlands are defined by two volcanic mountain ranges running from north to south. The botanical diversity of the region reflects in large measure variation in elevation: an inter-Andean valley of agricultural plots and pastures; lower montane rain forests and upper montane cloud forests on the mountain slopes; dry valleys in the south; and high altitude grass and desert *paramos *(plateaus) [6].

**Figure 1:**
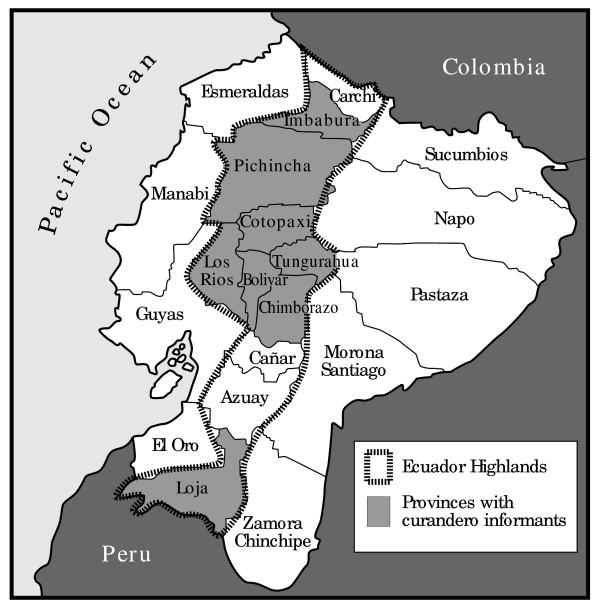
**Map of the Ecuador highlands and area surveyed**.

Figure 1 shows the area surveyed in terms of informant residence. As indicated in Table 1, most of the curanderos interviewed for this investigation resided in the south-central highlands, notably the Boliver, Chimborazo, and Tungurahura provinces. Table 1 also shows a strong bias in residence; the great majority of the informants (n = 85, 82%) resided in the Bolivar Province.

**Table 1:** Province residence of curanderos

**Province**	**Number**	**Percentage**
Bolivar	85	73.00

Chimborazo	10	8.60

Cotopaxi	3	2.50

Imbabura	1	.80

Los Rios	6	5.10

Pinchincha	1	.80

Tungurhua	10	8.62

Totals	116	100.00

Informant interviews were done in 2002 and 2003 as part of a more encompassing investigation on the role of curanderos in the region's informal health care delivery system. Published sources on ethnomedicine in the Ecuador highlands and the Andean region were used to develop an interview schedule to gather baseline information on: 1) recruitment to the curandero role, 2) years of service, 3) client load, 4) articulation with and attitude toward the formal health care system, 5) types of services rendered and healing specializations, 6) types of folk illnesses recognized and treated, 7) therapeutic methods and *materia medica *used in the treatment of folk illnesses, and 8) types of curanderos. The interview schedule was pre-tested with 14 informants and subsequently underwent minor modifications. The investigation was also informed by numerous unscheduled interviews with residents of various communities in the highlands and first-hand observation of ritual healings. This paper reports on information related to items six and seven on the interview schedule.

Initially, informants were recruited opportunistically through co-author Alban's contacts in the Bolivar and Chimborazo Provinces. Recruitment of informants proved difficult in the more isolated highlands communities due the authors' outsider status and their likely perceived identification with the government and the related concern of being reported for practicing medicine without a license. To overcome these and other recruitment problems, we solicited the assistance of second-year nursing students enrolled at the Universidad Estatal de Bolivar located in Guaranda. Our reasoning was that the students would be familiar with curanderos in their native communities and, more important, that the curanderos would be more inclined to do an interview with a resident of their community than with an outsider. At a meeting with the nursing students in 2003 a presentation was given by co-author Albán and the director of the university's nursing program on the purpose and scope of the project. The interview schedule and interviewing techniques were discussed later in classes with the students. The students were asked to identify and interview one curandero in their local community as a course assignment. Each student was given one dollar to cover transportation expenses. All informant responses were recorded directly on the interview schedule in Spanish. All the interviews, which on average lasted a little more than an hour, were done in the informants' homes. Since many informants were illiterate or marginally literate, written informed consent was not obtained. All informants, however, were orally informed of the purpose of the research and assured of the confidentiality of their identity. Not surprisingly, some students were much more successful than others doing the interview, and some informants were more prodigious and reflective in their responses than others. Eight interviews were withdrawn because the informants provided little or no information; four other interviews were withdrawn because they were informant duplicates.

**Figure 2:**
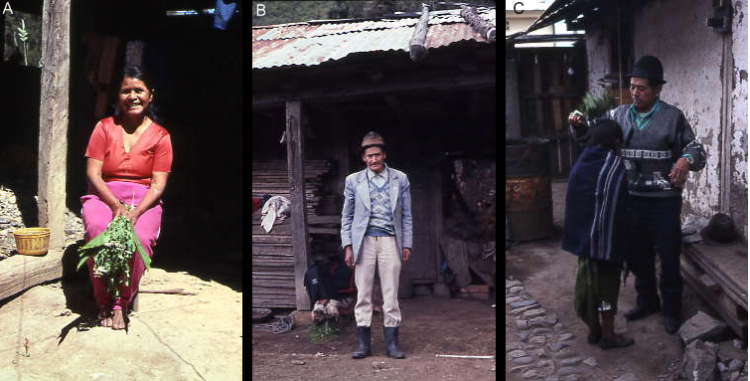
**Photos of selected Curanderos interviewed by this investigation**. A) Curandera from Chimbo, Bolivar Province, holding escobita used for brushing. B) Curandero from Cañi, Chimborazo Province. C) Curandero from Guanujo, Bolivar Province, brushing child.

Sixty-six (56.9%) of the curanderos were male, 50 (43.1%) were female. Of the 108 informants who reported their age, the range of age was 25 to 87 with an average age of 54. The majority (n = 67, 62%) were in the 40 to 59 age group; 10 were in the 20 to 39 age group. Years of service as a curandero ranged from 2 to 55 years with an average period of service of 21.57 years. Clients served by the informants per week ranged from 2 to 200 with an average client load of 21.9. Information on the level of educational attainment was obtained from 112 informants. Fifty-seven percent (n = 64) had completed primary school. Sixteen percent (n = 18) attended but did not complete primary school, and of these individuals 16 went no further than the fourth grade. Twenty-two percent (n = 25) were illiterate. Two informants completed high school and only one had a university degree. All the informants resided in rural communities, ranging from rural hamlets to small towns. The largest town represented in the study, Guaranda, has a population of about 5,000. Reflecting the rural economy of the highlands, the majority of the male informants worked as farmers or manual laborers. The female informants were mainly housewives, and most of them were actively involved in family farming activities. Some informants, both males and females, earned income through selling herbs, produce, animals and animal products, handicrafts, cooked foods, and merchandise at local town markets. If the client loads reported by the informants are valid, many curanderos enhanced their income considerably from their healing work. Some of their services are expensive. The performance of the *limpieza*, a cleansing ritual discussed later, can be, depending on a client's condition, depleting, costing as much as $25.00 according to some reports, and this does not include the cost of materia medica (e.g., cigarettes, cologne, plants, eggs, guinea pig) which in most cases the client is expected to bear. But even a few dollars is a lot for poor campesinos. (Photos of selected curanderos interviewed by this investigation are presented in Figures 2A–C)

As noted previously, all the informants were *limpiadores*, but many of them reported providing other much sought after services. Some informants were highly knowledgeable herbalists; some were *sobadores *(massage therapists); some were skilled at magically dealing with various social problems such as helping people with unfaithful husbands and wives, casting love spells, obtaining employment, finding lost objects, and assisting students with performing well on exams by magically treating their textbooks. Some informants, mainly but not exclusively females, also served as *parteras *(midwives). One informant served as a *llamador *("caller"), a healer who calls (i.e. retrieves) a departed soul back to an individual's body. The only type of curandero role not represented in the informant population is the shaman which we define as an individual who enters an altered state of consciousness to communicate with spirit beings. Shamans are present in the highlands, but are better represented in the Oriente (eastern rainforest) section of Ecuador.

Ethnicity in the highlands, which revolves around *mestizo*, *indígena *(indigenous, "Indian") and *blanco *(white) identities, is a sensitive and sometimes inflammatory topic for some residents. For this reason, information on ethnic self-identification was obtained only from informants who were friends or acquaintances of co-author Albán which amounted to 14. Eight self-identified as *mestizos*, 6 as *indígenas*. We compared the magical plants reported by the 12 *mestizo *curanderos with those reported by the 6 *indígena *curanderos and found a close correspondence in magical plants usage. Based on this admittedly limited comparison, we would predict that the belief in magical plants is a cultural tradition shared by both ethnic groups. A much more nuanced investigation, of course, might locate significant differences along ethnic lines.

## Results and discussion

The information on magical plants presented here is based on a content analysis of informant responses to the sections of the interview schedule concerning the types of folk illnesses recognized and related therapeutic methods and materia medica used to treat them. The analysis revealed the use of 48 plants and a variety of other materia medica for the treatment of *mal aire/mal viento, espanto/susto*, *brujería*, *duende*, and *mal prójimo*. This discussion, however, is confined to those plants (n = 17, 35%) that had a frequency of response of three or more. These plants and their uses are shown in Table 2. Unfortunately, no voucher specimens of the plants were collected. The scientific names for the plants in Table 2 were derived from cross-checking vernacular names with scientific names in four sources [4-7]. Final determination of the scientific name is based on *The Catalogue of Vascular Plants in Ecuador *[6]. The lack of identified voucher specimens, of course, presents problems in terms of determining accurately the correspondence of use of plants among the curandero informants. Furthermore, we were unable to infer from the vernacular names the taxanomical classification of two plants listed on Table 2 (manchari and tigresillo).

**Table 2:** Magical plants used by curanderos

**Scientific Name**	**Local Name**	**Reports**	**Part Used**	**Illness**	**Treatment Methods**
*Ambrosia arborecens* Mill. Asteraceae	marco	52	whole plant; leaves stems	mal aire (25) espanto (18) mal viento (9)	brush body with whole plant; infusion made from leaves ingested

*Baccharis latifolia* (Ruiz & Pav) Pers	chilca	13	whole plant	mal aire (7) espanto (4) mal prójimo	brush body

*Brugmansia sanguinea *(Ruiz & Pav.) D. Don Solanaceae	guanto rojo	8	leaves flowers	mal aire (5) mal viento (3)	brush body with leaves and flowers

*Citrus medica *L. Rutaceae	limón	6	fruit/rind	espanto (3) brujería (3)	rub body with whole fruit or rind

*Dianthus caryophyllus *L. Caryophyllaceae	clavel	12	flowers	mal aire (8) mal viento (4)	patient soaked in warm bath of flowers or sprayed with decoction of flowers

*Eucalyptus globulus *Labill. Myrtaceae	eucalipto	17	leaves stems	espanto (10) mal viento (5) mal prójimo(2)	brush body with small branches or vaporization of leaves

*Mentha *× *piperita *L. Lamiaceae	hierba buena	7	leaves	espanto (5) mal viento (2)	infusion ingested

*Minthostachys mollis *(Kunth) Griseb Lamiaceae	pumin	55	whole plant	mal aire (26) espanto (17) mal viento (12)	brush body with whole plant

*Nicotiana tabacum *L. Solanaceae	tabaco	50	leaves	mal aire (25) espanto (16) mal viento (6) duende (3)	smoke from cigarette blown on patient; also used to diagnose presence of *mal *(negative energy)

*Prunus serotina *Ehrh. Rosaceae	capulí	4	leaves stems	espanto (2) mal aire (2)	brushing with leaves

*Rosmarinus officinalis *L. Lamiaceae	romero	12	leaves stems	espanto (5) mal aire (4) mal viento (3)	brush body with leaves; infusion made from leaves and stems ingested

*Ruta graveolens *L. Rutaceae	ruda	65	whole plant; leaves, flowers	espanto (26) mal aire (26) mal viento (12) brujería (1)	brush with leaves; more rarely, patient soaked in warm bath of leaves and/or flowers

*Tanacetum parthenium *L. Sch. Bip Asteraceae	Santa María	46	whole plant	espanto (23) mal aire (17) mal viento (6)	brush with whole plant

*Urtica dioica *L. Urticaceae	ortiga negra	14	whole plant; leaves	espanto (7) mal aire (4) mal viento (3)	brush with whole plant; infusion made from leaves ingested

*Valeriana decussate *Ruiz & Pav. Valerianaceae	valeriana	6	leaves	espanto (3) mal viento (3)	infusion made from leaves ingested

Unknown	manchari	8	whole plant	espanto (8)	brush body with whole plant

Unknown	tigresillo	3	whole plant	mal aire (3)	brush body with whole plant

### Mal Aire/Mal Viento

Two authorities on ethnomedicine in Latin American observe that "airs" is "among the most elusive of illness concepts" [8]. Our investigation aligns with this observation. A few informants (n = 8) defined *mal aire *(bad air) as a naturalistic illness in terms of the hot/cold theory of illness causation. As they explained it, breathing in cold night air, moving rapidly from a warm to a cold ambient environment, or working up a sweat and not allowing for a proper cooling down period causes illness. The majority of informants, however, defined *mal aire *in supernatural terms. As for *mal viento *(bad or evil wind), several informants (n = 46, 39.6%) reported that the terms *mal aire *and *mal viento *are interchangeable and refer to the same kind of supernaturally caused illness. Although some informants recognized *mal aire *and *mal viento *as two distinct supernatural folk illnesses in terms of causation or symptoms, the definitional criteria used by some informants to distinguish *mal aire *were the very same criteria used by other informants to define *mal viento*.

The underlying supernatural meaning of *mal aire/mal viento *is the existence of a malevolent, destructive force or power that is transmitted through the air and wind. Following the discourse of some informants and McKee [9], we use the term *mal *in reference to this malevolent power. As observed by McKee in her study of diarrheal illness in the highlands, *mal *is in one sense animatistic, an impersonal force similar to the Melanesian concept of *mana*. Informants reported that *mal aire/mal viento *illness is caused by a person's exposure to *mal *at certain locales that they described as *lugares pesados *or *lugares malignos *(heavy or malignant places). These places include unpopulated areas, abandoned houses, gravesites, houses of the recently deceased, ravines, rock outcroppings, and gorges. They said that *mal *is naturally drawn to and gathers at these places, or that it is emitted by spirit beings that occupy these places. Upon contact *mal *sticks (*pega*) to a person and over a short period of time it penetrates and pollutes the entire body. Several informants said *mal *pollution results in internal decay (*decaimiento)*. If left untreated the illness is fatal. Children, notably unbaptized children, older adults, and physically debilitated persons are most vulnerable to *mal*. A constellation of symptoms are associated with *mal aire/mal viento*, manifesting in various combinations of coldness, diarrhea, headache, vomiting, paleness, fatigue, and shaking.

### Espanto/Susto

Like *mal aire/mal viento*, *espanto/susto *is a folk illness recognized in other parts of Latin America and among Latino populations in the United States [10-16]. Generally defined as magical fright, some investigations in the highlands report that *espanto *and *susto *are different illnesses [1,17], the former defined as soul loss caused by a frightening experience and the latter as milder form fright sickness. Most of the curanderos interviewed for this investigation, however, agreed that the two terms are synonomous and refer to an illness caused by a frightening experience that, if severe, results in soul loss. Infants and children are particularly susceptible. Some examples of causation shared by informants were an unexpected encounter with a wild animal, an uncommonly aggressive domestic animal, or a spirit being; scary dreams; an accident such as falling down, a car wreck, and a mishap with a tool; and a near accident such as almost being hit by a car or drowning. Receiving distressing news such as the death of a loved one was also reported. Informants explained that the departure of the soul is a dangerous circumstance because *mal *is attracted to and suffuses a souless body. Thus, as with *mal aire/mal viento*, a *limpieza *must be performed to clean the body of *mal *pollution. Symptoms of *espanto/susto *mentioned by informants varied but those most frequently mentioned were loss of appetite, vomiting, crying, sunken eyes, stomach ache, insomnia, and bad dreams.

### Mal prójimo, duende, and brujería

Other less frequently mentioned folk illnesses involving magical plant therapy mentioned in the interviews were *mal prójimo*, *duende*, and *brujería*. As described by informants and other investigators [1,3], *mal prójimo *("evil or bad neighbor") is an illness caused by the negative thoughts and feelings that a person or group of people has or have for another individual. The negative thoughts and feelings manifest as a destructive force or energy, described by some informants as *vibraciones *(vibrations) that harm the target individual. It is dynamically similar to another folk illness identified in the highlands known as *envidia *("envy") [1]. *Duende *("spirit") is an illness caused by an encounter with a spirit being that dwells in the countryside. *Brujería *(witchcraft) was mentioned by informants in the context of *brujos *(witches) manipulating negative energy to cause harm to others.

### La Limpieza

Etiologically, all the folk illnesses discussed above are conceptually linked to *mal *pollution. The function of the *limpieza *is to restore good health by cleansing *mal *from the body. The focus of this paper is the use of magical plants in the *limpieza *for *mal *removal, but it should be noted that informants mentioned a variety of non-plant materia medica used for the same purpose. These materials have been noted in other reports on the *limpieza *in the highlands [1,2] as well as other parts of Latin America [10,11,15]. The non-plant materials most frequently mentioned by informants include eggs, guinea pigs, *trago *(a sugar cane liquor, also known as *aguardiente*), holy water, candles, cologne, chickens, a sweaty shirt, and an over-the-counter bottled essence purchased at markets, "Seven Spirits." Some informants employ idiosyncratic power objects: a dog's skull, a doll's head, and dove's blood. As noted in other sources [1,2], the informants use eggs and guinea pigs for both diagnosing and curing folk illnesses. More often than not, the curanderos resort to a combination of plant and non-plant materials for the *limpezia*. The variety of combinations reported by the informants defies description.

A *limpieza *is performed when needed, but some informants indicated that it is most effective if done on a Tuesday or Friday between 6:00 p.m. and midnight. Depending on the degree of *mal *pollution, a *limpieza *may require several sessions. Curanderos first assess the physical symptoms and perform a diagnosis. Typically, a patient is asked to strip down to his underwear and a guinea pig or an egg is rubbed all over his body. Following the rubbing, the guinea pig, described by some informants as functioning like a *radiografía *(x-ray), is slit open ventrally while still alive and then splayed apart. The heart, liver, stomach, intestines, and lungs are examined. The extent of discoloration or disfiguration of the organs indicates the kind of illness. Eggs are cracked open in a glass of water and examined in terms of buoyancy in the water, coloration, and configuration. Others blow cigarette smoke on the arms and legs and observe whether the smoke adheres closely to the skin. If it does, then *mal *pollution is evident.

### Magical Plant Characteristics and Uses

It appears that the primary shared characteristic of the plants listed in Table 2 is odoriferousness. As explained by the informants, it is the scent of leaves or flowers of plants like chilca (*Baccharis obtusifolia*), pumin (*Minthostachys mollis*), clavel (*Dianthus caryophylis*), eucalipto (*Eucalyptus globulus*), marco (*Ambrosia arborecens*), capulí (*Prunus serotina*), hierba buena (*Mentha *× *piperita*), Santa María (*Tanacetum parthenium*), romero (*Rosmarinus officinalis*), and ruda (*Ruta graveolens*) that attracts and draws *mal *from within the body to the surface of the skin and into the plant. Though less frequently mentioned, the fruit or rind of limón (*Citrus medica*) is also used because of its strong odor. More often than not, these plants are used topically. The most common method is to take branches of one or more plants and weave them together to form a whisk broom variously called an *escobita *or *ramos benditos *(holy branches). Some curanderos warm the broom over a fire to pull out a plant's scent. The patient is then brushed with the broom from head to foot. Other methods of topical administration were reported. Some curanderos rub the leaves of plants together in their hands or crush them in a bowl to enhance the scent and then rub their scented hands or the crushed plants on the patient. Others said that they make a decoction of the plants by boiling them in a pot and then spraying the decoction on the patient by mouth or with a tube. A related method involves placing the patient on a mat, sitting or standing, and pouring or spraying the decoction on him and then rubbing the decoction into the skin. A few informants reported immersing the patient in a bath of clavel flowers. Infusions are also employed, ostensibly for forcing *mal *out of the body. Plants used as infusions include the aforementioned hierba buena, romero, and marco, but also valeriana (*Valeriana decussata*). Having the patient inhale vapors from a hot decoction of eucalipto leaves was mentioned by some informants as another method for forcing *mal *out of the body. As noted earlier, tobacco smoke (*Nicotiana tabacum*) from a cigarette is used for diagnosis, but it is also thought to be effective for pulling *mal *out of the body. Smoke is blown all over a patient's body.

The notion of something odorous drawing *mal *from the body is evident in other materia medica used in a *limpieza *such as cologne and the over-the-counter popular magic product mentioned earlier, "Seven Spirits," both of which are sprayed by mouth on the patient's body. It is also illustrated in one curandero's use of a sweaty shirt. He told a story of a client who said that he had been unexpectedly hit by a *huracán *(dust devil or twister) while working in a field. The informant and the client agreed that the *huracán *was the manifestation of a spirit and that the client was polluted with *mal*. Treatment entailed the curandero running about his farm until he produced a sweat soaked shirt smelling strongly of body odor which he used to rub down his client's body.

Informants were not asked if the magical plants used in the *limpieza *are *caliente *(hot), *fresca *(cold) or *templada *(neither hot nor cold), but according to Kothari's [18] and Lombeyda's [5] research on medicinal plants in the highlands (the Imbabura and Bolivar Provinces, respectively), 10 (58.8%) of the 17 plants listed in Table 2 are *caliente*, two are *fresca*, one is *templada*, and four are not classified. Further research might identify a humoral determinant in the selection of plants for treating folk illnesses.

Magical plants are not only used for cleaning people of *mal *pollution, but also for the *sahumerio*, a ritual used to remove *mal *from a house. Informants said that a *sahumerio *is performed when a family moves into a house that has been abandoned for a lengthy period of time, when someone has died in the house, and when a family has experienced an abnormal number of misfortunes like crop failure, death of livestock, death of relative, loss of employment, and accidents. The house is cleaned by burning incense and/or sweeping the floors, walls, and ceilings of a house with plants like Santa Maria, marco, chilca, and ruda.

The plants used in a *limpieza *are gathered in the wild, purchased in local markets, or, as observed by Finnerman and Sackett [19], some (e.g., clavel, hierba buena., romero, and ruda) are cultivated in *huertas *(home gardens). It should be noted that, though curanderos are considered more capable of performing a *limpieza *because they have *el don *(the gift) or *el mano bueno *(the good hand), lay people also perform the *limpieza *at home, especially for children [4].

Following a *limpezia*, the plants and other materials used for cleaning are carefully disposed since they contain harmful *mal *and are viewed much like radioactive and medical waste. One must dispose of them in a place far removed from human habitation and concourse to prevent *mal *contagion of others. As observed by McKee [9], *mal *cannot be destroyed; it is gradually released back into nature from the healing materials that temporarily contained it.

## Conclusion

The treatment of folk illnesses constitutes the major part of the practices of the curanderos interviewed by this investigation. All of them reported that they perform the *limpieza *for the treatment of *espanto/susto, mal aire/mal viento*, *mal prójimo*, *duende*, and *brujería*. Physicians are not capable of diagnosing and treating these illnesses; only a *limpiador *can.

Of the magical plants reported, only one, guanto rojo (*Datura sanguinea*), belongs to the hallucinogenic category of magical plants, but it is not ingested. Notably absent among the hallucinogenic magical plants indigenous to the highlands is San Pedro cactus (*Echinopsis pachanoi*), an integral element of the culturally similar Quechua ethnomedical tradition in northern Peru [20]. This plant can be gathered in some parts of the Ecuador highlands or purchased in some of the local markets. Recent research on medicinal plants in the Loja Province in the southern portion of the highlands by Bussman and Sharon [4] noted the absence of its use among curanderos there. They speculate that it was likely used in the past but was abandoned due to its prohibition by the government and church, but we know of no evidence of the use of San Pedro in the highlands during or prior to the conquest period. Other hallucinogenic magical plants not mentioned by the informants include several species indigenous to the Oriente (rainforest) section of Ecuador such as *Banisteriopsis caapi *and *Psychotria viridis*. In conversations with residents of the highlands, several said that the Oriente has many plants with *más poder *(more power), referring to the plants' hallucinogenic properties. According to Saloman [21], the visionary and curing tradition of the shamans in the Oriente has been held in awe by the highlands residents for hundreds of years. Some of the hallucinogenic plants from the Oriente are available in local markets in the highlands and through traveling herb dealers that make a yearly circuit encompassing the highlands and the Oriente [22], but none of the curanderos interviewed for this study employ them. This absence is perhaps attributable to no shamans being interviewed for this investigation.

Understood within the context of their use, magical plants reveal the central, single-most important integrative concept of the supernatural component of *curanderismo *in the Ecuador highlands: *el mal*. (Note again that many of the residents interviewed by this study did not use this term. Other terms used to identify the same concept include "energy," "power," and "force." Following McKee [9], we elected to use the term *mal*.) The concept is directly connected to the etiology and treatment of a wide array of folk illnesses, not only in the Ecuador highlands but also in other parts of Central and South America [8,12-16]. It is unclear if the concept of *el mal *is derived from Spanish or indigenous sources, but it is apparent that both mestizo and indigenous residents of the highlands believe in *el mal *and the efficacy of the *limpieza*. In her study of ethnomedicine in Guaranda, a predominantly mestizo town in the highlands, Lombeyda [5] observes that many of the better educated mestizos publically dismiss curanderos as ignorant illiterates and witches while at the same time secretly using their services. We know several mestizos as well as some who claim a blanco identity, including physicians, who frequently seek the help of curanderos for a *limpieza *and other services. On another level, the *mal *concept provides a portal into the mindscape of some highlanders, i.e., the symbolic transmutation of the physical features of the landscape into places that are topophobically perceived as charged with dangerous animate and inanimate forces. These places are akin to what cultural geographers call "landscapes of fear" [23], "sick places" [24], or "landscapes of despair" [25]. This topophobic aspect has been identified among other mestizo and indigenous populations in the Ecuador highlands [1-3,17,26], Bolivia [27], and Peru [28]. It is a mindscape deeply rooted in what historian MacCormack [29] calls the "sacred topography" of the pre-Christian, Incan, and pre-Incan era, a time when "the plains and the mountains, the sky and the waters were both the theatre and the dramatis personae of divine action (p.146)." She notes that for many contemporary Andeans, "earth and sky are still inhabited by the ancient powers" [p. 433]. As noted by anthropologist George Foster [11], the highlanders' mindscape was also influenced by beliefs from Spain concerning dangerous "airs" emitted from gravesites and evil spirits riding the wind.

We do not intend to impart the notion that the residents of the highlands live in a constant state of fear of malevolent forces; rather, it is recognition of a living landscape that the highlanders must carefully navigate and negotiate. Conversely, there are therapeutic landscapes in the highlands, places symbolically associated with health and healing such as cathedrals, shrines, lakes, and waterfalls. Furthermore, there is the countervailing and overarching Quichua (or in Peru, Quichua) concept of *pacha mama*, the vital force of "mother nature" that suffuses and sustains all living things [30,31]. For Catholic highlanders, God does the same. Thus, it is the vital, benevolent power of *pacha mama *or God that flows through the magical plants used by humans to defend themselves against the debilitating and sometimes fatal effect of *mal*.

## Competing interests

The authors declare that they have no competing interests.

## Authors' contributions

Both authors contributed to the fieldwork and data analysis. AC is responsible for writing the manuscript.
